# Role of Abdominal Contrast-Enhanced Computed Tomography in Diagnosing Gangrenous Gall Bladder Wall Perforation

**DOI:** 10.7759/cureus.107958

**Published:** 2026-04-29

**Authors:** G Swathi, Manasa Reddy, Sundararajan Srinivasan

**Affiliations:** 1 Department of Radiology, Meenakshi Medical College Hospital and Research Institute, Meenakshi Academy of Higher Education and Research, Chennai, IND

**Keywords:** complicated acute cholecystitis, contrast-enhanced ct, gallbladder perforation, gangrenous cholecystitis, mural enhancement defect, pericholecystic abscess

## Abstract

Background: Complicated acute cholecystitis, particularly gangrenous cholecystitis and gallbladder perforation, represents a radiologic and surgical emergency because delayed recognition may lead to sepsis, abscess formation, and worse operative outcomes. Although ultrasonography is the first-line imaging modality, its ability to detect mural necrosis and perforation remains limited. This study evaluated the role of contrast-enhanced computed tomography (CECT) in identifying advanced gallbladder inflammation in patients with surgically confirmed complicated disease.

Methods: This retrospective observational study was conducted over a two-year period after ethics approval. Adult patients with clinically suspected acute or complicated cholecystitis who underwent contrast-enhanced CT abdomen and subsequently had operative and histopathological confirmation were included. Twenty patients with complete clinical, radiological, operative, and pathological records constituted the final cohort.

Results: The mean age was approximately 54 years, with 11/20 males (55.0%) and 9/20 females (45.0%). Right upper quadrant pain was the most common presenting feature, seen in 19/20 patients (95.0%), followed by abdominal tenderness in 16/20 (80.0%), leukocytosis in 16/20 (80.0%), fever in 14/20 (70.0%), nausea or vomiting in 13/20 (65.0%), and positive Murphy's sign in 12/20 (60.0%). On ultrasonography, gallstones were identified in 16/20 (80.0%), gallbladder wall thickening in 15/20 (75.0%), distension in 9/20 (45.0%), pericholecystic fluid in 6/20 (30.0%), and suspicion of perforation in 4/20 (20.0%). On CT, wall thickening was present in 17/20 (85.0%), irregular wall contour in 12/20 (60.0%), pericholecystic fluid in 10/20 (50.0%), absence of mural enhancement in 9/20 (45.0%), intraluminal membranes in 6/20 (30.0%), gas in the gallbladder wall or lumen in 3/20 (15.0%), and wall discontinuity in 8/20 (40.0%). Surgery confirmed gangrenous cholecystitis in 12/20 patients (60.0%) and gallbladder perforation in 8/20 (40.0%).

Conclusion: Contrast-enhanced CT served as a decisive problem-solving modality in complicated acute cholecystitis by identifying mural devascularization, gangrene, perforation, and extra-gallbladder complications more reliably than ultrasonography. Its major value lies in pattern-based recognition of advanced disease that may directly influence operative urgency and treatment planning.

## Introduction

Acute cholecystitis is usually diagnosed on the basis of clinical findings and ultrasonography, yet the real radiologic challenge begins when inflammation progresses beyond uncomplicated disease into gangrenous cholecystitis or gallbladder perforation [1,2]. These advanced forms are not merely morphologic variants; they represent ischemic necrosis and structural failure of the gallbladder wall, often accompanied by abscess formation, biliary contamination, sepsis, and a greater need for urgent operative management. Contemporary evidence has repeatedly shown that delay in recognizing these complications worsens outcomes [3]. In a broad evidence synthesis, Fabbri et al. (2025) [4] emphasized that severe forms of acute cholecystitis benefit from timely surgical treatment, while Derici et al. (2006) [5] and Gunasekaran et al. (2015) [6] showed that perforation remains diagnostically elusive and carries substantial morbidity and mortality. From a radiologic standpoint, the problem is not the visibility of inflammation itself, but the reliable preoperative identification of gangrene, mural devascularization, and frank perforation before catastrophic progression occurs.

Ultrasonography remains the first-line imaging modality because it is rapid, inexpensive, and sensitive for gallstones, wall thickening, and pericholecystic fluid. However, its performance becomes less dependable in complicated disease, where the key issue is not merely confirming cholecystitis but defining severity and identifying wall necrosis or disruption. Simeone et al. (1989) [7] demonstrated that the sonographic Murphy's sign, usually highly informative in ordinary acute cholecystitis, may be absent in many patients with gangrenous disease, likely reflecting denervation of the necrotic wall. Derici et al. (2006) [5] likewise reported that ultrasonography failed to demonstrate a gallbladder wall defect in perforation cases, even when indirect findings were present. This diagnostic blind spot is clinically important because the radiologist may encounter a patient whose ultrasound appears compatible with acute cholecystitis, while the true pathology is already gangrenous or perforated. That gap explains why cross-sectional imaging, especially contrast-enhanced computed tomography (CECT), has become increasingly important in patients with suspected severe or atypical biliary inflammation.

Contrast-enhanced CT offers a broader anatomic survey and a more direct evaluation of the gallbladder wall, surrounding fat planes, adjacent liver, peritoneal cavity, and extra-gallbladder complications. Prior studies have identified several CT features that should heighten suspicion for gangrenous transformation, including irregular or absent wall, reduced mural enhancement, marked distension, intraluminal membranes, mural striation, gas within the wall or lumen, and pericholecystic abscess [8-10]. For perforation, the most decisive sign remains focal wall discontinuity, supported by pericholecystic collection, abscess, biloma, free fluid, or hepatic extension [11-13]. Even so, the literature also makes clear that CT is not infallible: some signs are highly specific but insufficiently sensitive, and many studies are retrospective with relatively small cohorts. Thus, there remains a practical need for institution-level data that correlate contrast-enhanced CT findings with operative and histopathological confirmation in real-world patients with suspected complicated cholecystitis.

Against this background, the present retrospective observational study was undertaken to evaluate the role of contrast-enhanced CT abdomen in adults with clinically suspected complicated acute cholecystitis who ultimately had surgical confirmation of gangrenous cholecystitis or gallbladder perforation.

## Materials and methods

This retrospective observational study was conducted in the Departments of Radiology and General Surgery at Meenakshi Medical College Hospital and Research Institute after Institutional Ethics Committee approval (MMCHRI IEC/PG/08/NOV/24). Hospital records collected over a two-year period were reviewed to evaluate the role of CECT of the abdomen in patients with suspected complicated acute cholecystitis, with particular emphasis on gangrenous cholecystitis and gallbladder perforation.

Study population

Adult patients aged 18 years and older who presented with clinical suspicion of acute cholecystitis or complicated acute cholecystitis and underwent CECT abdomen were screened for inclusion. Clinical suspicion was based on symptoms and signs such as right upper quadrant abdominal pain, fever, nausea or vomiting, abdominal tenderness, positive Murphy's sign, and laboratory evidence of inflammation, including leukocytosis. To maintain consistency with the final analyzed cohort, only patients with complete clinical, radiological, operative, and histopathological records were included. The final study population consisted of 20 patients who underwent surgery and had operative confirmation of gangrenous cholecystitis or gallbladder perforation.

Inclusion and exclusion criteria

Inclusion criteria were age of 18 years or older, clinical suspicion of acute or complicated cholecystitis, performance of CECT abdomen for further evaluation, and availability of operative and histopathological confirmation following cholecystectomy. Patients were excluded if records were incomplete, if they had other gallbladder pathologies such as gallbladder carcinoma without perforation, or if they had contraindications to iodinated contrast administration, including severe renal impairment. These criteria were applied to ensure consistency between imaging findings and final surgical diagnosis.

Data collection

Data were retrieved from hospital case records, the radiology information system, operative notes, and histopathology reports. The collected variables included age, sex, comorbidity status, presenting symptoms, leukocytosis, ultrasonographic findings, CECT findings, operative findings, and final histopathological diagnosis. Ultrasonographic variables included gallstones, gallbladder wall thickening, gallbladder distension, pericholecystic fluid, and suspicion of perforation. CECT variables included gallbladder wall thickening, irregular wall contour, reduced or absent mural enhancement, intraluminal membranes, gas within the gallbladder wall or lumen, pericholecystic fluid, focal wall discontinuity, pericholecystic abscess, free intraperitoneal fluid, localized bile collection, and hepatic abscess. These variables were selected to correspond directly with the findings reported in the Results section.

CT acquisition protocol

All contrast-enhanced CT examinations were performed on a multidetector CT scanner in the radiology department. Scans were obtained with the patient in the supine position after intravenous administration of iodinated contrast through a peripheral venous cannula using an automated injector, with the dose adjusted according to body weight. Image acquisition included an arterial phase at approximately 20 to 30 seconds and a portal venous phase at approximately 60 to 70 seconds after contrast administration. Scan coverage extended from the dome of the diaphragm to the pelvis. Thin-section axial images were acquired, and multiplanar reconstructions in the coronal and sagittal planes were generated for detailed evaluation of the gallbladder, surrounding fat planes, and adjacent abdominal structures.

Image interpretation

CT images were reviewed by experienced radiologists with expertise in abdominal imaging. The scans were assessed for gallbladder wall thickness, wall contour abnormality, focal wall discontinuity, mural enhancement pattern, intraluminal membranes or debris, gas within the gallbladder lumen or wall, pericholecystic fluid, pericholecystic abscess, free intraperitoneal fluid, localized bile collection, hepatic abscess, and inflammatory changes in adjacent tissues. The predefined CT features assessed were gallbladder wall thickening, irregular wall contour, focal wall discontinuity, reduced or absent mural enhancement, intraluminal membranes, gas within the wall or lumen, pericholecystic fluid, pericholecystic abscess, localized bile collection, free intraperitoneal fluid, and hepatic abscess. When discrepant interpretations arose, the final imaging assessment was established by consensus review.

Reference standard

The final diagnosis was established using operative findings at cholecystectomy, with histopathological examination of the excised gallbladder specimen used as confirmatory evidence. Operative and histopathological records were reviewed for necrosis, gangrenous change, inflammatory infiltration, and perforation of the gallbladder wall. Final diagnoses were categorized as gangrenous cholecystitis or gallbladder perforation in accordance with the operative outcomes.

Statistical analysis

Data were entered and analyzed using IBM SPSS Statistics for Windows, Version 29.0 (IBM Corp., Armonk, NY). Continuous variables were summarized using mean and range, while categorical variables were expressed as frequency and percentage. Because the present study was a retrospective single-cohort descriptive analysis with a small sample size, statistical analysis was primarily descriptive. Ultrasonographic findings, CECT findings, and operative diagnoses were summarized as proportions.

## Results

Baseline demographic and clinical characteristics

A total of 20 patients with clinically suspected complicated acute cholecystitis who underwent contrast-enhanced CT of the abdomen were included in the study. The age of the patients ranged from 28 to 78 years, with a mean age of approximately 54 years. There were 11 male patients (55.0%) and nine female patients (45.0%). Diabetes mellitus was present in seven patients (35.0%) and hypertension in six patients (30.0%), while seven patients (35.0%) had no documented comorbidity. The original wording suggesting that all 20 patients had gangrenous perforation was inconsistent with the final surgical breakdown and has therefore been corrected in this cleaned version to remain numerically aligned with the reported operative diagnoses (Table 1).

**Table 1:** Baseline demographic characteristics of the study population (n = 20) Values are presented as n (%). This table summarizes the age distribution, sex distribution, and comorbidity profile of the 20 patients included in the study.

Variable	n (%)
Age group	-
21-30 years	1 (5.0)
31-40 years	3 (15.0)
41-50 years	5 (25.0)
51-60 years	6 (30.0)
>60 years	5 (25.0)
Sex	-
Male	11 (55.0)
Female	9 (45.0)
Comorbidity status	-
Diabetes mellitus	7 (35.0)
Hypertension	6 (30.0)
No comorbidity	7 (35.0)

Clinical presentation

Right upper quadrant pain was the most common presenting symptom and was documented in 19 patients (95.0%). Abdominal tenderness and leukocytosis were each observed in 16 patients (80.0%). Fever was present in 14 patients (70.0%), nausea or vomiting in 13 patients (65.0%), and a positive Murphy's sign in 12 patients (60.0%) (Table 2).

**Table 2:** Clinical presentation of the study cohort (n = 20) Values are presented as n (%). This table shows the frequency of major presenting symptoms and laboratory-associated clinical findings in the study cohort.

Clinical feature	n (%)
Right upper quadrant pain	19 (95.0)
Fever	14 (70.0)
Nausea/vomiting	13 (65.0)
Abdominal tenderness	16 (80.0)
Leukocytosis	16 (80.0)
Positive Murphy's sign	12 (60.0)

Ultrasonography findings

Ultrasonography was performed as the initial imaging modality in all patients. Gallstones were identified in 16 patients (80.0%) and gallbladder wall thickening in 15 patients (75.0%). Gallbladder distension was noted in nine patients (45.0%), while pericholecystic fluid was observed in six patients (30.0%). Suspicion of perforation on ultrasonography was documented in four patients (20.0%) (Table 3).

**Table 3:** Ultrasonography findings (n = 20) Values are presented as n (%). This table summarizes the initial ultrasonographic findings recorded in the 20 patients evaluated for suspected complicated cholecystitis.

Ultrasonography finding	n (%)
Gallstones	16 (80.0)
Gallbladder wall thickening	15 (75.0)
Distended gallbladder	9 (45.0)
Pericholecystic fluid	6 (30.0)
Suspicion of perforation	4 (20.0)

CECT abdomen findings

Contrast-enhanced CT of the abdomen was performed in all 20 patients. Gallbladder wall thickening was the most frequent CT finding and was seen in 17 patients (85.0%). Irregular wall contour was present in 12 patients (60.0%), while pericholecystic fluid was detected in 10 patients (50.0%). Absence of mural enhancement was observed in nine patients (45.0%), intraluminal membranes in six patients (30.0%), and gas within the gallbladder wall or lumen in three patients (15.0%) (Table 4).

**Table 4:** CECT abdomen findings in the study cohort (n = 20) Values are presented as n (%). This table summarizes the principal CECT findings identified in patients with suspected complicated acute cholecystitis.

CT finding	n (%)
Gallbladder wall thickening	17 (85.0)
Irregular wall contour	12 (60.0)
Pericholecystic fluid	10 (50.0)
Absence of mural enhancement	9 (45.0)
Intraluminal membranes	6 (30.0)
Gas in gallbladder wall/lumen	3 (15.0)

CT findings related to gallbladder perforation

Among CT features related to perforation, wall discontinuity or the hole sign was identified in eight patients (40.0%). Pericholecystic abscess was present in five patients (25.0%), and free intraperitoneal fluid in four patients (20.0%). Localized bile collection was observed in two patients (10.0%) and hepatic abscess in one patient (5.0%) (Table 5).

**Table 5:** CT findings suggestive of gallbladder perforation (n = 20) Values are presented as n (%). This table presents the direct and indirect CT features associated with gallbladder perforation in the study cohort.

CT feature	n (%)
Wall discontinuity (hole sign)	8 (40.0)
Pericholecystic abscess	5 (25.0)
Free intraperitoneal fluid	4 (20.0)
Localized bile collection	2 (10.0)
Hepatic abscess	1 (5.0)

Surgical confirmation

**Figure 1:**
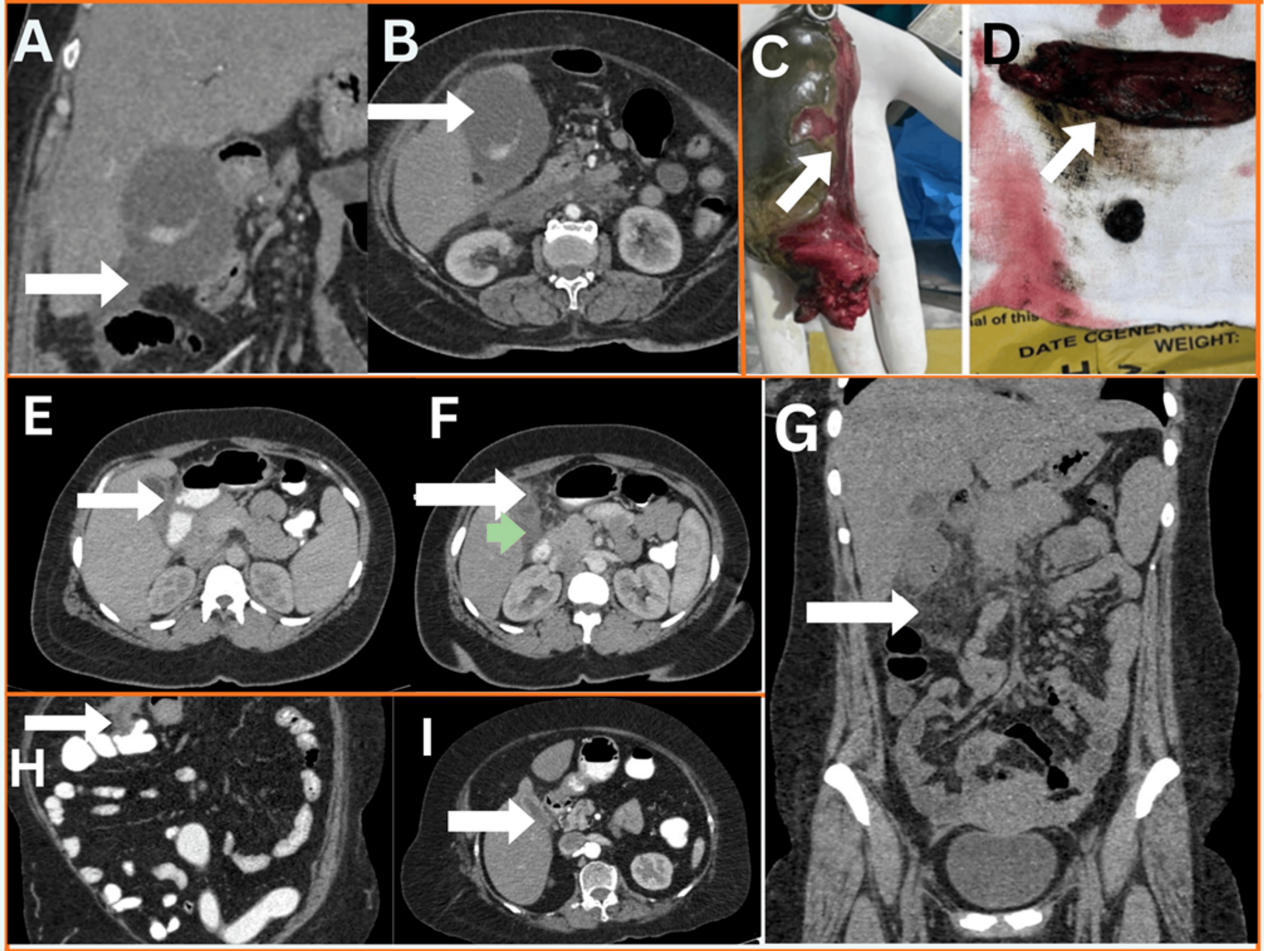
Contrast-Enhanced Computed Tomography and Gross Pathologic Correlates of Complicated Acute Calculous Cholecystitis A: Coronal computed tomography image showing a gallbladder calculus (white arrow). B: Axial contrast-enhanced computed tomography image showing pericholecystic fluid (white arrow). C: Gross specimen showing gangrenous gallbladder with necrosis and focal perforation (white arrow). D: Gross specimen showing hemorrhagic gangrenous tissue (white arrow). E: Axial contrast-enhanced computed tomography image showing irregular gallbladder wall thickening with focal fundal defect (white arrow). F: Axial contrast-enhanced computed tomography image showing adjacent pericholecystic collection (white arrow) and surrounding inflammatory change (green arrow). G: Coronal contrast-enhanced computed tomography image showing pericolonic and mesenteric fat stranding adjacent to the gallbladder region (white arrow), consistent with sealed-off perforation. H: Coronal contrast-enhanced computed tomography image showing adherence of the transverse colon to the gallbladder with loss of fat plane (white arrow). I: Axial contrast-enhanced computed tomography image showing focal discontinuity and non-enhancement of the gallbladder wall (white arrow).

All 20 patients underwent surgical intervention. Intraoperative confirmation documented gangrenous cholecystitis in 12 patients (60.0%) and gallbladder perforation in eight patients (40.0%). These operative findings constituted the final confirmed diagnoses in the cohort (Table 6). Representative CECT appearances with gross pathologic correlation of complicated acute calculous cholecystitis are shown in Figure 1.

**Table 6:** Surgical confirmation of final diagnosis (n = 20) Values are presented as n (%). This table summarizes the final operative diagnosis in all patients included in the study.

Final diagnosis	n (%)
Gangrenous cholecystitis	12 (60.0)
Gallbladder perforation	8 (40.0)
Total confirmed surgically	20 (100.0)

## Discussion

The present study reinforces a clinically important radiologic message: in suspected complicated acute cholecystitis, contrast-enhanced CT contributes far more than mere confirmation of inflammation; it helps expose the transition from simple mural inflammation to ischemia, necrosis, and structural failure of the gallbladder wall. In this cohort of 20 surgically confirmed cases, gangrenous cholecystitis constituted the larger subgroup at 60.0%, while gallbladder perforation accounted for 40.0%. On CT, gallbladder wall thickening was the commonest finding, but the more meaningful markers of complicated disease were irregular wall contour, absent mural enhancement, intraluminal membranes, gas, and especially wall discontinuity in perforated cases. By contrast, ultrasonography, although useful as the initial test, raised suspicion of perforation in only 20.0% of patients, underscoring its limited ability to define severity once the disease becomes necrotic or perforated.

These findings are broadly concordant with prior radiologic literature. Bennett et al. (2002) showed that CT signs such as irregular or absent wall, absent enhancement, intraluminal membranes, gas, and pericholecystic abscess are highly specific for gangrenous cholecystitis, even though their sensitivity is limited [8]. Sureka et al. (2018) similarly emphasized that no single sign is diagnostic, but the constellation of wall irregularity, distension, reduced enhancement, mural striation, and intraluminal membranes strongly favors gangrenous transformation [9]. Chang et al. (2016) further strengthened the value of decreased mural enhancement and gallbladder distension as specific indicators of gangrene [10]. The current study mirrors that pattern well: wall thickening alone was common but nonspecific, whereas irregular contour, absent enhancement, and membranes carried greater interpretive weight in identifying severe disease. In practical terms, the present data support the view that CT is most useful when interpreted as a pattern-recognition tool rather than as a search for one pathognomonic sign.

The perforation findings are also in agreement with established evidence. Derici et al. (2006) [5], Kim et al. (1994) [14], Harraz and Abouissa (2020) [12], and Tsai et al. (2009) [13] all identified mural wall defect or wall discontinuity as the most decisive CT sign of perforation, usually accompanied by pericholecystic collection, abscess, biloma, free fluid, or hepatic extension. In the present study, the hole sign was seen in 40.0% of all patients, and among the indirect accompaniments were pericholecystic abscess, free intraperitoneal fluid, localized bile collection, and hepatic abscess. This pattern closely matches the pathophysiologic reality of perforation: once mural integrity is breached, imaging findings extend beyond the gallbladder and begin to map the route of bile leakage and secondary infection. The results therefore support the continuing centrality of CT in preoperative mapping of perforated disease, especially when ultrasonography remains equivocal.

At the same time, the study also echoes an important limitation noted in the literature: complicated cholecystitis does not always declare itself with dramatic imaging features. Gas in the wall or lumen, for example, was present in only 15.0% of cases here, despite being regarded by Bennett et al. (2002) as extremely specific [8]. Similarly, absent mural enhancement was observed in less than half of the cohort, meaning that its absence cannot exclude gangrene. This partial discordance with some reports is not surprising. First, the present cohort combined both gangrenous cholecystitis and perforation rather than isolating a single phenotype. Second, the sample size was small, which magnifies percentage variation. Third, CT appearances are heavily influenced by timing: a scan obtained before complete devascularization or before frank rupture may show only evolving changes. Fourth, institutional differences in contrast timing, image quality, and threshold for surgery may alter the frequency with which “classic” signs are captured. Thus, the current results support a nuanced conclusion already advanced by prior authors: CT findings in complicated cholecystitis are highly informative, but they must be interpreted in a clinical context rather than used in isolation as binary rule-in or rule-out markers.

The clinical implications are direct. In patients with right upper quadrant pain, fever, leukocytosis, and an ultrasound that demonstrates stones or wall thickening but does not convincingly explain the severity of illness, CT should not be viewed as a redundant second test. Instead, it should be deployed as the modality that defines complication burden, identifies mural ischemia, detects perforation, and alerts the surgeon to pericholecystic abscess or extra-gallbladder spread. This is particularly relevant because delayed recognition of gangrenous cholecystitis and perforation has been associated with greater morbidity and mortality, while timely surgery improves outcomes, as emphasized by Fabbri et al. (2025) [4], Derici et al. (2006) [5], and Gunasekaran et al. (2015) [6]. In radiologic practice, the reporting language should therefore move beyond “acute cholecystitis” when features such as irregular wall, reduced enhancement, membranes, gas, or wall discontinuity are present; such cases warrant explicit communication of suspicion for gangrene or perforation.

This study has limitations that must be acknowledged. It was retrospective, single-center, and included only 20 surgically confirmed patients, which limits generalizability and prevents robust inferential analysis. Because the study included only surgically and histopathologically confirmed complicated cases, the cohort represents a highly selected population, which may limit reproducibility and generalizability to the wider spectrum of patients with suspected acute cholecystitis. As only operated and confirmed complicated cases were included, the study cannot estimate sensitivity, specificity, or predictive values against uncomplicated controls. The absence of blinded independent rereading and the descriptive statistical approach also restricts formal diagnostic performance assessment. Nevertheless, the study retains value because it correlates real-world CT findings directly with operative confirmation in a clinically consequential subgroup.

## Conclusions

In this retrospective cohort of surgically confirmed complicated acute cholecystitis, contrast-enhanced CT demonstrated characteristic findings associated with gangrenous change and gallbladder perforation, particularly wall irregularity, absent mural enhancement, intraluminal membranes, gas, and wall discontinuity. Ultrasound remained useful for initial evaluation, whereas CT provided additional morphologic characterization of advanced disease and associated complications. These findings should be interpreted as descriptive and hypothesis-generating, and larger comparative studies are needed to define the diagnostic performance of CT across the broader spectrum of suspected acute cholecystitis.
